# Modeling mortality prediction in older adults with dementia receiving COVID-19 vaccination

**DOI:** 10.1186/s12877-024-04982-7

**Published:** 2024-05-24

**Authors:** Zorian Radomyslsky, Sara Kivity, Yaniv Alon, Mor Saban

**Affiliations:** 1 Maccabi Healthcare Services, 6812509 Tel Aviv-Jaffa, Israel; 2 Ariel University, School of Health Sciences, Ariel, Israel; 3 Nursing Department, School of Health Professions, Faculty of Medical & Health Sciences, Tel Aviv University, Ramat Aviv, Tel Aviv, 69978 Israel

**Keywords:** Cognitive impairment, Older adults, COVID-19 vaccination, Mortality, Predictive analytics

## Abstract

**Objective:**

This study compared COVID-19 outcomes between vaccinated and unvaccinated older adults with and without cognitive impairment.

**Method:**

Electronic health records from Israel from March 2020-February 2022 were analyzed for a large cohort (*N* = 85,288) aged 65 + . Machine learning constructed models to predict mortality risk from patient factors. Outcomes examined were COVID-19 mortality and hospitalization post-vaccination.

**Results:**

Our study highlights the significant reduction in mortality risk among older adults with cognitive disorders following COVID-19 vaccination, showcasing a survival rate improvement to 93%. Utilizing machine learning for mortality prediction, we found the XGBoost model, enhanced with inverse probability of treatment weighting, to be the most effective, achieving an AUC-PR value of 0.89. This underscores the importance of predictive analytics in identifying high-risk individuals, emphasizing the critical role of vaccination in mitigating mortality and supporting targeted healthcare interventions.

**Conclusions:**

COVID-19 vaccination strongly reduced poor outcomes in older adults with cognitive impairment. Predictive analytics can help identify highest-risk cases requiring targeted interventions.

**Supplementary Information:**

The online version contains supplementary material available at 10.1186/s12877-024-04982-7.

## Introduction

The coronavirus disease 2019 (COVID-19) pandemic created immense challenges for older adults with Alzheimer's disease and related dementias (ADRD) [1, 2]. These progressive neurological conditions involve impairment of cognitive functions including memory, language, and thinking, which were exacerbated by the pandemic's disruptions [3]. Indeed, individuals with ADRD have faced disproportionate adverse outcomes from COVID-19 infection compared to those without dementia, including higher mortality rates as shown in meta-analyses [4]. Increased morbidity, accelerated cognitive and functional decline, and a rise in urgent hospitalizations have also been reported in this population [5, 6]. These negative effects resulted from reduced access to formal caregivers, unemployment among individuals with ADRD, and physical isolation during lockdowns [2, 7]. Disrupted routines and decreased activity levels also contributed to documented cases of worsened behavioral issues and depression [6].

Accurately predicting mortality risk is especially critical for patients with ADRD, as it allows clinicians to have informed discussions with families, guide treatment decisions, and provide appropriate levels of care [7, 8]. However, while COVID-19 vaccinations have benefited the general senior population, their precise impacts on outcomes among older adults with ADRD remain less understood [2, 5]. Despite full vaccination, older adults with ADRD have shown higher breakthrough infection risks than vaccinated seniors without dementia [9]. Accordingly, there is a need for research comparing mortality rates and hospitalization incidence between vaccinated and unvaccinated individuals with ADRD across all pandemic phases. This study utilized predictive modeling to compare long-term mortality and hospitalization rates between people with and without ADRD, differentiated by vaccination status. These advanced analytics were employed since they can offer insights into vaccination effectiveness for this vulnerable group, and generate valuable prognostic information to aid patients, families, and providers.

## Methods

This was a retrospective cohort study of the electronic medical records between March 01, 2020 and February 28, 2022, of older adults aged ≥ 65 living in the community. All participants were insured for at least two years prior to the study [T1 (2018–2020)] and during the study time frame [T2 (2020–2022)] with Maccabi Healthcare Services (MHS), one of the major health management organizations in Israel. The cohort was divided into a group of patients with dementia and an age, gender, and socioeconomically matched control group without dementia. Both patient groups were further divided into those who had received COVID-19 vaccination and those who had not (see below).

### Data collection

The electronic medical records for the dementia study group were obtained from the Cognitive Disorders Registry established by MHS in 2019. This registry was designed to facilitate comprehensive monitoring of patients with cognitive deterioration and includes the continuum of patients with pre-dementia mild cognitive impairment (MCI) all the way to those with severe dementia. The data for the control group was obtained from electronic medical records in MHS's general national database. The primary independent variable was COVID-19 vaccination status. Patients who had received a minimum of two mRNA or viral vector vaccine doses were designated as vaccinated, while patients who had received either a sole dose or none were designated as non-vaccinated. This classification leveraged the vaccination dates as a cumulative factor, ensuring a dynamic assessment of vaccination status over time.Data was collected on sociodemographic variables, including age, gender, geographical location, and socioeconomic strata; COVID-19 characteristics including COVID-19 infection and number of vaccine doses; and clinical attributes including severity of dementia, prescription or utilization of antipsychotic and/or antidepressant medications, diagnosed depression, utilization of home-based medical regimens, incidents of bone fractures including hip fractures, and the presence of documented medical conditions spanning hypertension, chronic obstructive pulmonary disease (COPD), diabetes mellitus (DM), immunosuppression, and obesity.

Data on healthcare-associated variables were also obtained, including metrics related to activities of daily living (ADL); incidences of visits to the emergency department; cumulative hospitalization duration; frequency of hospitalizations, geriatric clinic visits, family-centered clinic visits, geriatric teleconsultations, family telephonic interactions, appointments with psychiatrists, and social worker consultations; frequency of falls; and their attendant costs.

### Data processing

**Fig. 1:**
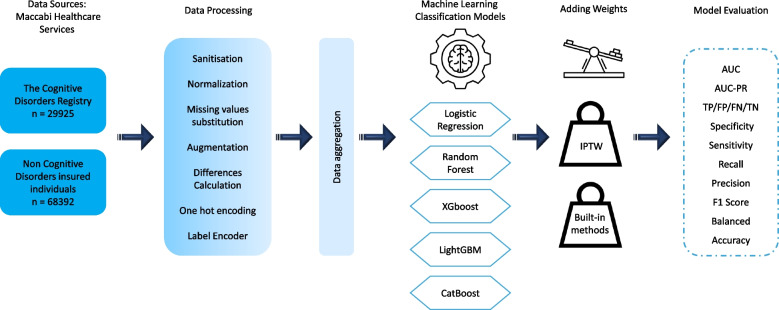
Data integration and pre-processing pipeline for integrated databases

In the data processing pipeline (Fig. 1), several fundamental steps were undertaken to ensure the quality, reliability, and suitability of the dataset for subsequent analysis. The process began with sanitization, involving the cautious removal or modification of sensitive, redacted, or inconsistent entries to enhance data integrity. Subsequently, normalization was employed to standardize data features to a uniform scale, enabling equitable comparisons and analyses among diverse attributes. Addressing the challenge of missing values, a substitution strategy was applied, wherein gaps in the dataset were filled using techniques like K-Nearest Neighbors (KNN) imputation or predefined placeholders. To augment the dataset's richness and diversity, augmentation methods were deployed, generating new instances through replication, combination, or duplication of existing data points. Computation of absolute differences played a significant role for clinical characteristics, facilitating the calculation of variations between periods T1 (2018–2020) and T2 (2020–2022). Categorical data underwent transformation through one-hot encoding, converting categorical variables into numerical format by creating binary columns. Another approach, label encoding, was employed to assign unique integer labels to categorical categories. Data aggregation was carried out to merge datasets from diverse sources, such as healthcare records, vaccination records, and demographic information. This process facilitated a comprehensive view of individual profiles by combining relevant attributes into a unified record for each subject. These data pre-processing steps collectively ensured data readiness for subsequent analysis, enhancing the reliability and validity of insights derived from the dataset in the context of the study's objectives.

### Statistical analysis

Descriptive statistics, including means and standard deviations for continuous variables, and percentages for categorical variables, were utilized to characterize the sociodemographic characteristics, COVID-19 characteristics, and clinical characteristics of the participants. To assess the statistical differences between groups, T-tests were employed for continuous variables, while Chi-squared tests were used for categorical variables, as appropriate. The level of significance for all statistical analyses was 5%. The data analysis was performed using Python (version 3.9.16).

### Machine learning models

For mortality prediction, we employed both logistic regression and tree-based models (Fig. 1). In our utilization of the logistic regression model, we adopted the "newton-cg" solver, which leverages second-order derivative information (Hessian matrix) to adjust its search direction. This characteristic contributes to quicker convergence, especially when addressing imbalanced data scenarios. The "newton-cg" solver is particularly suited for imbalanced datasets exhibiting distinct class separation. This suitability is observed when a majority of instances within the minority class are distinctly separated from those in the majority class [10–13].

In refining our classifier tree-based models, including Random Forest, XGBoost, LightGBM, and CatBoost, we employed a comprehensive approach to hyperparameter tuning using random search cross-validation, as facilitated by the scikit-learn library. This process was particularly focused on optimizing the Area Under the Curve (AUC) as the primary loss function. Following this, we leveraged the best-performing hyperparameters to evaluate the models on a separate validation set. Our evaluation function not only computed the AUC-ROC and AUC-PR but also employed bootstrapping to estimate 95% confidence intervals for these metrics, providing a robust assessment of model performance. This approach enabled us to address potential overfitting effectively and ensure the generalizability of our predictive models across unseen data, as demonstrated through detailed metrics including recall, precision, F1 score, and balanced accuracy. Our methodology thus underscores the rigor of our model fitting and validation process, ensuring the reliability and applicability of our findings to broader contexts. The hyperparameters that underwent adjustment included the number of trees (n_estimators), the maximum depth of trees (max_depth), and the learning rate. It's important to note that there were some differences for specific classifiers. For instance, the learning rate was not applicable to the Random Forest classifier, and for the CatBoost classifier, the maximum depth was constrained to a maximum value of 16, leading to the use of different intervals during tuning. With these exceptions, the majority of hyperparameters were consistent across models. To determine the best model, an evaluation metric called the area under the receiver operating characteristic curve (AUC score) was utilized. The same process of fine-tuning was extended to models that incorporated various built-in methods to address class imbalance, as detailed in Table 2. In addition to these methods, a technique called inverse probability of treatment weighting (IPTW) was employed in conjunction with the logistic regression and Gaussian Naive Bayes (GaussianNB) algorithms [14].

IPTW was used to counteract the bias introduced by the imbalanced distribution of mortality in the dataset. This was achieved by assigning appropriate weights to individual instances based on their treatment propensities. In essence, these weights represented the degree to which each instance was representative of the overall population. Logistic regression and GaussianNB were employed to model the propensity scores, which indicate the likelihood of an individual being vaccinated. These propensity scores were then utilized to compute weights for each instance in the dataset. The strength of this methodology lies in the combination of logistic regression and GaussianNB to compute propensity scores, leading to the generation of customized weights for each instance. These individualized weights were then used to counteract the adverse effects of class imbalance in the dataset. The ultimate result was an enhancement in both the fairness and accuracy of subsequent predictions related to mortality. The assessment of the models involved the utilization of diverse scoring techniques provided by the scikit-learn library. These methods encompassed AUC, as discussed above, area under the precision recall (AUC-PR), confusion matrix, specificity, sensitivity, recall, precision, F1 score, and balanced accuracy. Furthermore, the evaluation of the random search cross-validation encompassed the utilization of the learning_curve function. This function facilitated the determination of cross-validated training and test scores, specifically across varying training set sizes. To visualize the models' performance, their AUC and AUC-PR values were portrayed using the roc_curve and precision_recall_curve functions from the scikit-learn library. This allowed for a comprehensive understanding of how the models' predictive capabilities were distributed across different thresholds and recall-precision balances. The AUC-PR metric focuses its evaluation on the performance of the positive (minority) class, thus enabling a more targeted assessment of a model's effectiveness in identifying and classifying instances of the minority class, which is often of greater interest in imbalanced scenarios. Confidence intervals (CIs) for both AUC and AUC-PR were computed to quantify the uncertainty associated with these performance metrics. Bootstrapping, a resampling technique, was employed to generate multiple resampled datasets from the original data, allowing for the creation of distributions of AUC and AUC-PR values. By repeating this process numerous times, confidence intervals were established by determining the lower and upper bounds of percentiles within the distributions. These intervals provided a measure of the range within which the true AUC and AUC-PR values were likely to fall with a specified confidence level [15].

### Ethical consideration

The study protocol was approved by the Institutional Human Subjects Ethics Committee of Maccabi Healthcare Services (0075–22-MHS) of the relevant medical facility. Written informed consent was waived by the Institutional Review Board of Maccabi Healthcare Services. All performed procedures followed the ethical standards of both the institutional and national research committees.

## Results

In the study, 29,925 patients were ascribed to the Cognitive Disorder Registry and 68,392 apart of the control group. Out of the remaining individuals, 68,556 (80%) had received vaccination, while 16,732 (20%) remained unvaccinated. Throughout the duration of the study, the Cognitive Disorder Registry was associated with 29,925 individuals, and 68,392 individuals were included in the control group, totaling 98,317 individuals in the cohort. To ensure the integrity and comparability of our data, 13,029 individuals were excluded during the data pre-processing phase. This exclusion was due to the absence of presence in both periods, T1 (before the pandemic) and T2 (during the pandemic), rather than solely based on unmatched criteria like age, gender, and socioeconomic status. This approach was taken to accurately assess the impact of COVID-19 vaccination on mortality by comparing individuals with consistent data across both time frames.Statistical analysis revealed a significant difference in gender, age, and socioeconomic status (SES) distribution between the vaccinated and unvaccinated groups (*p* < 0.001), making the unvaccinated group older and with a lower SES (Appendix 1). This demographic disparity was due to a larger number of vaccinated patients in the study sample. During the initial sampling, vaccination status was not considered as a parameter, and the two groups initially showed no significant differences in demographic characteristics.

A higher proportion of the vaccinated individuals (37%) tested positive for COVID-19 compared to the unvaccinated group (18%) (*p* < 0.001). However, the unvaccinated group exhibited a significantly higher mortality rate (51%) compared to the vaccinated group (3%) (*p* < 0.001), emphasizing the role of vaccination in protecting against mortality. The unvaccinated individuals also showed a higher prevalence of cognitive disorders (38% vs. 25%, *p* < 0.001), dementia characteristics (8% vs. 6%, *p* < 0.001), immunosuppression (28% vs. 18%, *p* < 0.001), diabetes (37% vs. 33%, *p* < 0.001), use of antipsychotics medication (24% vs. 12%, *p* < 0.001), and prescription rates for psychiatric medication (17% vs. 11%, *p* < 0.001).

The unvaccinated group also had other disadvantages to the vaccinated group including more hospitalization days (see Table 1).

**Table 1:** Continuous variables analysis. Mean and standard deviation (SD) of the absolute magnitudes of differences between the measurements taken at time points T2 and T1

	Unvaccinated		Vaccinated		
	**mean**	**SD**	**mean**	**SD**	***p* values (t test)**
**Applications to the emergency department difference**	0.65	1.34	0.56	0.97	< 0.001
**Hospitalization days difference**	18.07	46.92	6.03	24.91	< 0.001
**Number of hospitalizations difference**	2.24	3.43	1.13	1.86	< 0.001
**Frontal geriatrics visits difference**	0.28	1.23	0.22	1.00	< 0.001
**Frontal family visits difference**	11.94	14.61	10.06	10.31	< 0.001
**Geriatric visits phone difference**	0.14	1.29	0.07	0.94	< 0.001
**Family visits phone difference**	0.42	2.49	0.16	1.52	< 0.001
**Psychiatrist visits difference**	0.17	0.90	0.19	1.11	0.08
**Social worker visits difference**	1.07	2.91	0.68	2.47	< 0.001
**Amount of falls difference**	0.54	2.26	0.54	2.19	0.9
**Cost difference (ILS)**	46,588.89	86,085.54	27,033.27	57,209.83	< 0.001
**ADL difference**	2.24	1.58	1.89	1.56	< 0.001

These discrepancies in the continuous variables resulting from the data imbalance between the vaccinated and unvaccinated patients could significantly affect the training of the machine learning models to predict mortality. The imbalance in the distribution of these variables may introduce bias and skew the model's learning process, making it more challenging to accurately predict mortality outcomes for both the vaccinated and unvaccinated groups. To tackle the imbalance, the machine learning models were categorized into four distinct groups: those without weighted classes, models utilizing class-weighting techniques, models incorporating IPTW using logistic regression, and models employing IPTW with GaussianNB (as outlined in eAppendix 2).

**Fig. 2:**
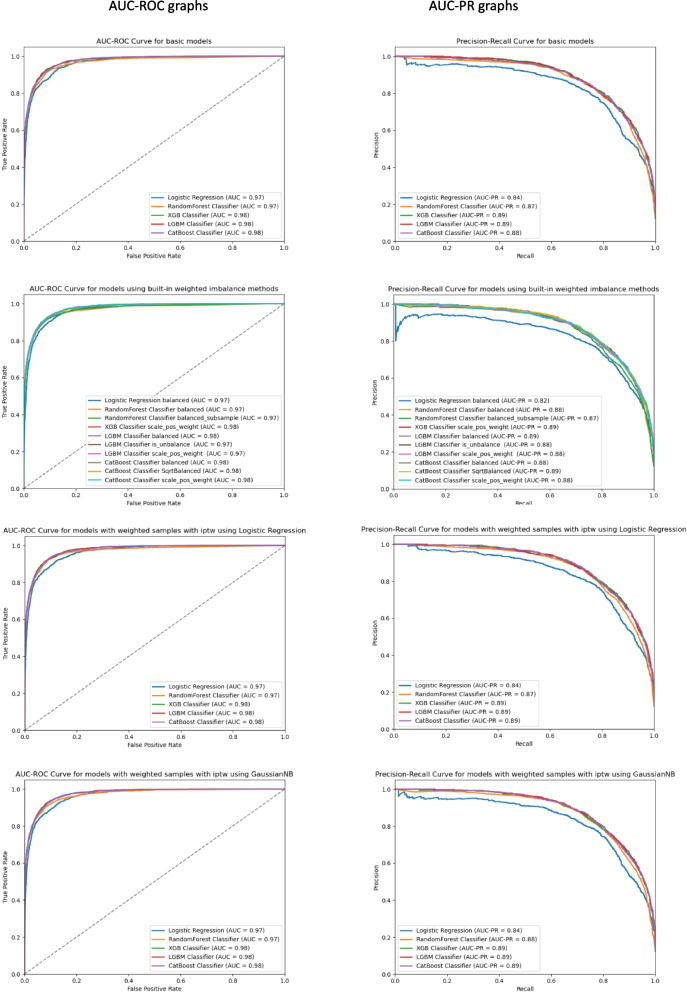
The Effect of Imbalance Weighting and IPTW on the performance of machine learning models for Binary mortality prediction

The models were rigorously evaluated on the validation set using a comprehensive suite of scoring metrics, with a particular focus on AUC and AUC-PR curves, as illustrated in Fig. 2. All models, each employing distinct weighting methodologies, consistently exhibited AUC values ranging from 0.96 to 0.98, along with AUC-PR values between 0.82 and 0.89, indicative of robust performance. Nevertheless, LightGBM, when employing IPTW with GaussianNB, attained the highest AUC value, while XGBoost, devoid of weighted methods, yielded the highest AUC-PR value, positioning them as potential optimal models. Despite the promising scores, the prospect of overfitting necessitated attention. While several models demonstrated elevated AUC scores across all sections, certain models displayed overfitting tendencies when comparing training and validation results using the learning_curve method. Notably, the random forest and CatBoost models exhibited overfitting, with random forest displaying the most severe case. Strikingly, the logistic regression method showcased excellent outcomes concerning overfitting, evidenced by the minimal disparity between training and validation AUC values. The results for XGBoost also indicated convergence between training and validation AUC values. While LightGBM demonstrated promising training and validation AUC outcomes, it showed comparatively limited generalization capabilities compared to XGBoost and logistic regression. To identify the optimal model for the test set, confidence intervals (CIs) for both AUC and AUC-PR were employed. The XGBoost model utilizing IPTW calculated with logistic regression exhibited the narrowest AUC-CI and AUC-PR-CI (AUC CI: 0.97624–9, AUC-PR CI: 0.8903–5), suggesting enhanced confidence in its performance assessment. The test set outcomes, using the XGBoost model utilizing IPTW calculated with logistic regression, resulted with an AUC value of 0.9773 and an AUC-PR value of 0.8969. The AUC CI (0.97732–0.97737) and AUC-PR CI (0.8969- 0.8971) further substantiate the model's reliable performance.

The test set confusion matrix [TP/FP/FN/TN 12484/207/456/1352, respectively] reiterated the model's capacity in effectively distinguishing between true positives, false positives, true negatives, and false negatives (Table 2).

**Table 2:** Evaluation metrics test set results of XGBoost Model using IPTW with logistic regression

**AUC [95%CI]**	0.9773 [0.97732–0.97737]
AUC-PR [95%CI]	0.8969 [0.8969–0.8971]
TP/FP/FN/TN	[12484, 207, 456, 1352]
Specificity	0.7772
Sensitivity	0.9543
Recall	0.7478
Precision	0.8672
F1 Score	0.8031
Balanced Accuracy	0.8657

**Fig. 3:**
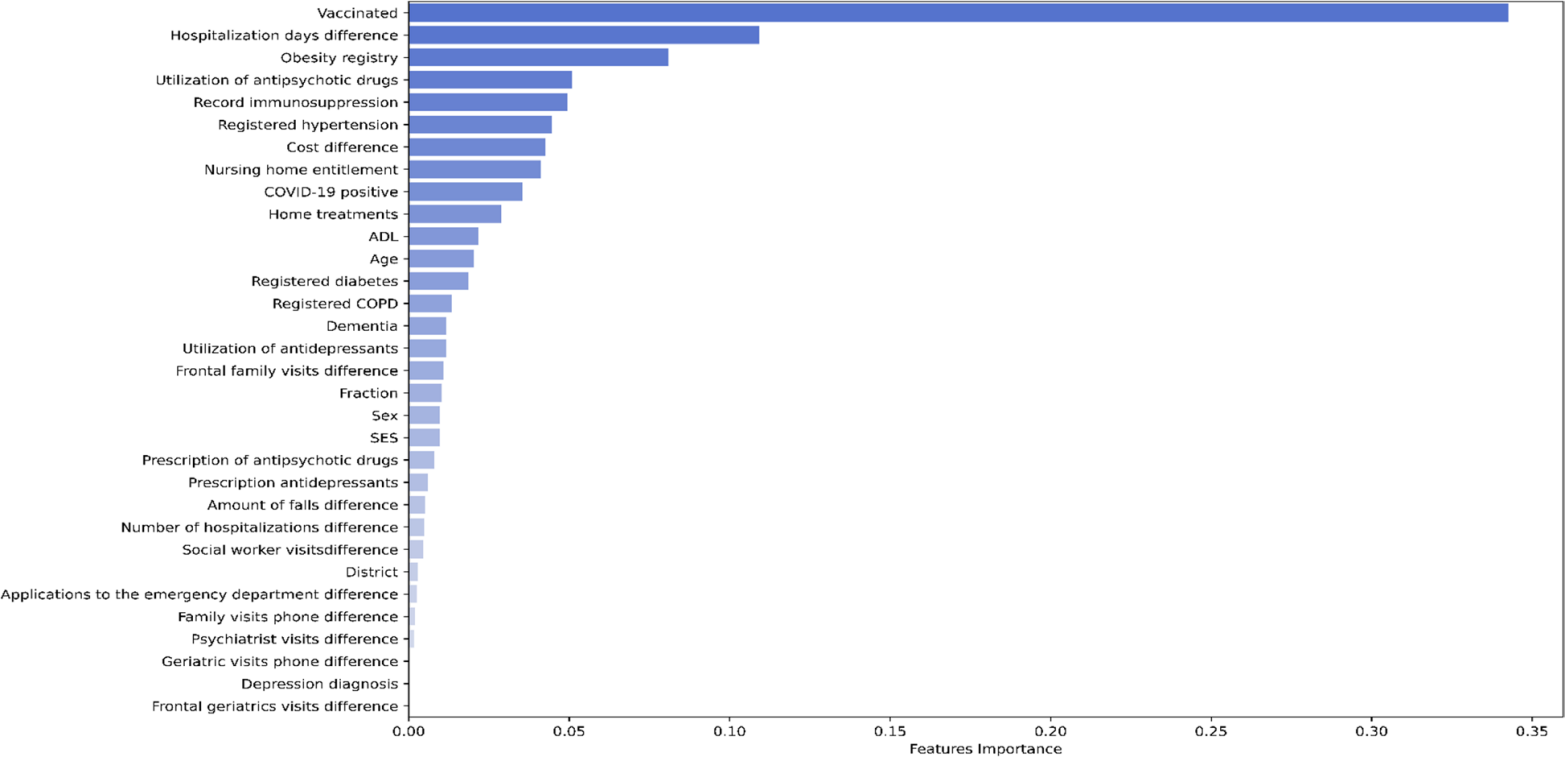
Feature importance analysis within the XGBoost model with IPTW weights calculated using logistic regression

The feature importance analysis from the selected model (Fig. 3) underscores the substantial influence of the "Vaccinated" feature in predicting mortality within the population with cognitive disorders, including dementia. The relatively high feature importance value assigned to "Vaccinated" (0.34) implies a notable association between COVID-19 vaccination and a potentially protective effect against mortality in this specific group. This finding suggests that individuals who have been vaccinated against COVID-19 may experience a reduced likelihood of mortality compared to their unvaccinated counterparts within the context of cognitive disorders and related health conditions. Additionally, the feature importance attributed to "Hospitalization days difference" (0.11) within the predictive model holds noteworthy implications for mortality prediction which indicates that changes in hospitalization duration, possibly reflecting the severity of medical conditions or healthcare interventions, are closely associated with mortality risk. Therefore, individuals with shorter hospitalization stays or those who experience more substantial fluctuations in their hospitalization durations may exhibit distinct mortality patterns. Among cognitive disorder related features 'Dementia' holds a relatively higher importance (0.01175) indicating that it contributes more significantly to mortality prediction compared to 'prescription of antipsychotic drugs' (0.008), 'utilization of antidepressants' (0.0117), 'prescription of antidepressants' (0.006), and 'depression diagnosis' with zero influence. A comprehensive analysis unveiled that when specifically investigating the significance of the "Vaccinated" feature within the subgroup of individuals registered with cognitive disorders, its importance is notably higher at 0.65, as opposed to those individuals not included in the Cognitive Disorder Registry, where its importance measures 0.42. While other comorbidities had a varying feature importance, the analysis of feature importance revealed a finding concerning the impact of treatment-related factors, encompassing costs, home treatment, nursing home entitlement, and ADL values. This collective influence indicates that the presence or absence of attentive care, whether through these treatment measures, strongly influences mortality prediction. Moreover, the observation that various visiting features exhibit low to negligible importance in mortality prediction suggests potential factors at play. This phenomenon could be attributed to the context of the COVID-19 period, during which individuals may not have maintained regular visits to healthcare practitioners. Additionally, the proximity of a caregiver or lack thereof for individuals above the age of 65 emerges as a compelling determinant in influencing individual longevity, potentially overshadowing the significance of other visiting features Table 3.

**Table 3:** Performance Comparison of Various Machine Learning Models for Binary Mortality Prediction. Description: Description: The table displays performance metrics for various machine learning models employed in binary mortality prediction on the validation set. Evaluation criteria include AUC, AUC-PR, confusion matrix (TP/FP/FN/TN), specificity, sensitivity, recall, precision, F1 score, and balanced accuracy. The models encompass fundamental machine learning algorithms, models employing built-in weighted imbalance methods, and models utilizing inverse probability of treatment weighting, incorporating both Logistic Regression and GaussianNB

Model	Weighted method	AUC [95% CI]	AUC-PR [95% CI]	TP/FP/FN/TN	Specificity	Sensitivity	Recall	Precision	F1 Score	Balanced Accuracy
Logistic Regression	None	0.9665 [0.9665–0.9665]	0.8393 [0.8393–0.8396]	12,117/272/500/1269	0.7499	0.9455	0.7174	0.8235	0.7668	0.8477
RandomForest	None	0.9701 [0.97–0.9701]	0.8779 [0.8777–0.8779]	12,205/184/500/1269	0.7508	0.9517	0.7174	0.8734	0.7877	0.8513
XGBoost	None	0.9763 [0.9762–0.9763]	0.8905 [0.8903–0.8905]	12,176/213/474/1295	0.7634	0.9515	0.7321	0.8588	0.7904	0.8574
LightGBM	None	0.9757 [0.9757–0.9757]	0.8889 [0.8887–0.8889]	12,166/223/466/1303	0.7672	0.9513	0.7366	0.8539	0.7909	0.8593
CatBoost	None	0.9751 [0.9751–0.9751]	0.8849 [0.8849–0.8851]	12,202/187/511/1258	0.7453	0.9507	0.7111	0.8706	0.7828	0.8480
Logistic Regression	class_weight = 'balanced'	0.9673 [0.9673–0.9673]	0.824 [0.824–0.8243]	11,072/1317/163/1606	0.9061	0.8955	0.9079	0.5494	0.6846	0.9008
RandomForest	class_weight = 'balanced'	0.9672 [0.9672–0.9673]	0.8698 [0.8696–0.8699]	12,204/185/523/1246	0.7394	0.9500	0.7044	0.8707	0.7788	0.8447
RandomForest	'balanced_subsample'	0.9698 [0.9698–0.9699]	0.8771 [0.877–0.8772]	12,208/181/520/1249	0.7410	0.9505	0.7060	0.8734	0.7809	0.8457
XGBoost	scale_pos_weight	0.9759 [0.9759–0.9759]	0.8858 [0.8857–0.8859]	11,270/1119/134/1635	0.9224	0.9115	0.9243	0.5937	0.7230	0.9170
LightGBM	class_weight = 'balanced'	0.9757 [0.9756–0.9757]	0.8852 [0.8851–0.8853]	11,315/1074/139/1630	0.9204	0.9143	0.9214	0.6028	0.7288	0.9174
LightGBM	is_unbalance	0.9749 [0.9748–0.9749]	0.8821 [0.8819–0.8821]	11,310/1079/136/1633	0.9218	0.9142	0.9231	0.6021	0.7289	0.9180
LightGBM	scale_pos_weight	0.9749 [0.9748–0.9749]	0.8821 [0.882–0.8822]	11,310/1079/136/1633	0.9218	0.9142	0.9231	0.6021	0.7289	0.9180
CatBoost	auto_class_weights = 'balanced'	0.9751 [0.9751–0.9751]	0.8821 [0.882–0.8822]	11,271/1118/138/1631	0.9205	0.9113	0.9220	0.5933	0.7220	0.9159
CatBoost	auto_class_weights = 'SqrtBalanced'	0.9755 [0.9754–0.9755]	0.8866 [0.8866–0.8868]	11,966/423/327/1442	0.8340	0.9470	0.8151	0.7732	0.7936	0.8905
CatBoost	scale_pos_weight	0.9751 [0.9751–0.9752]	0.8821 [0.882–0.8822]	11,271/1118/138/1631	0.9205	0.9113	0.9220	0.5933	0.7220	0.9159
Logistic Regression	LogisticRegression propensity weighted	0.9652 [0.9651–0.9652]	0.8411 [0.841–0.8413]	12,114/275/511/1258	0.7445	0.9445	0.7111	0.8206	0.7620	0.8445
RandomForest	LogisticRegression propensity weighted	0.9696 [0.9696–0.9697]	0.8767 [0.8766–0.8769]	12,210/179/519/1250	0.7415	0.9507	0.7066	0.8747	0.7817	0.8461
XGBoost	LogisticRegression propensity weighted	0.9762 [0.9762–0.9762]	0.8901 [0.89–0.8902]	12,182/207/477/1292	0.7620	0.9517	0.7304	0.8619	0.7907	0.8568
LightGBM	LogisticRegression propensity weighted	0.9759 [0.9759–0.9759]	0.8875 [0.8874–0.8876]	12,181/208/476/1293	0.7624	0.9517	0.7309	0.8614	0.7908	0.8571
CatBoost	LogisticRegression propensity weighted	0.9754 [0.9754–0.9754]	0.8877 [0.8876–0.8878]	12,194/195/480/1289	0.7606	0.9523	0.7287	0.8686	0.7925	0.8565
Logistic Regression	GaussianNB propensity weighted	0.9655 [0.9655–0.9656]	0.8351 [0.8351–0.8354]	12,094/295/495/1274	0.7522	0.9442	0.7202	0.8120	0.7633	0.8482
RandomForest	GaussianNB propensity weighted	0.9699 [0.9699–0.97]	0.8774 [0.8773–0.8775]	12,212/177/501/1268	0.7504	0.9521	0.7168	0.8775	0.7890	0.8513
XGBoost	GaussianNB propensity weighted	0.9761 [0.9761–0.9761]	0.8886 [0.8886–0.8888]	12,188/201/488/1281	0.7566	0.9513	0.7241	0.8644	0.7881	0.8540
LightGBM	GaussianNB propensity weighted	0.9763 [0.9763–0.9764]	0.8904 [0.8903–0.8904]	12,176/213/463/1306	0.7688	0.9523	0.7383	0.8598	0.7944	0.8605
CatBoost	GaussianNB propensity weighted	0.9755 [0.9755–0.9756]	0.8888 [0.8887–0.8889]	12,199/190/476/1293	0.7626	0.9530	0.7309	0.8719	0.7952	0.8578

## Discussion

The key findings of this study offer important insights into the effects of COVID-19 vaccination on mortality outcomes in older adults with cognitive disorders and dementia. Among this high-risk population, a lack of vaccination was associated with dramatically increased risks of mortality, despite higher COVID-19 positivity rates in the vaccinated group. These results align with previous studies demonstrating reduced mortality after vaccination in older and medically fragile populations [16–18].

For dementia specialists, the findings of a strong protective effect of COVID-19 vaccination on mortality risk among those with cognitive impairment have broader clinical implications as we enter the post-pandemic period. Continuing to emphasize the significance of regular immunization against high-risk respiratory pathogens, such as influenza and COVID-19, offers clinicians the opportunity to extend the observed advantages in longevity that emerged during the pandemic to this particularly susceptible patient demographic [19]. The optimization of vaccination coverage stands as a potent, yet underutilized avenue for diminishing preventable mortality and morbidity linked with vaccine-preventable diseases among individuals with dementia [19, 20].

The considerable imbalance between vaccinated and unvaccinated groups introduces confounding biases reflecting real-world demographic disparities in vaccination uptake. The unvaccinated individuals tended to be older, frailer, and sicker, with higher rates of comorbid conditions like DM and immunosuppression that could independently increase mortality risk [21, 22]. The unvaccinated group also had greater needs for healthcare services including hospitalization and daily living assistance. By employing rigorous statistical weighting techniques during modeling, our machine learning approach accounted for these imbalances. The feature importance analyses further adjust for factors like dementia severity when identifying vaccination as the top predictor of mortality [23, 24].

The feature importance analysis from the optimized XGBoost model illuminates the contribution of various factors in predicting mortality risk within this vaccinated and unvaccinated cohort of older adults with cognitive disorders. The prominent role of the "Vaccinated" feature, especially within those with cognitive disorders, highlights a potential protective association between COVID-19 vaccination and reduced mortality in this high-risk group [8, 25]. Hospitalization days difference by any case also emerged as an influential indicator of mortality, implying that greater disease severity and more intensive healthcare interventions are closely linked to worse outcomes [26, 27].

The collective significance of treatment-related factors, such as costs, access to home care, eligibility for nursing home services, and assistance requirements for activities of daily living, underscores the pivotal role of comprehensive supportive care in shaping mortality outcomes, suggesting that greater care availability promotes longevity in this population [28, 29].

Proximity of caregivers emerged as a salient predictor of mortality specifically among those over 65 years of age. This highlights the key role that regular in-person care and assistance from dedicated caregivers may play in promoting longevity in older populations, including those with cognitive impairment [30–32]. On the other hand, visiting features related to frequency of visits from physicians, nurses and social workers carried little importance in the predictive model. This lack of significance for visiting features likely stems from widespread limitations or reductions in routine in-person healthcare visits during the COVID-19 pandemic period. With temporary disruptions to normal healthcare access, the frequency of visiting various providers was likely diminished and may not have held its typical influence in mortality prediction models [33]. However, this finding underscores the need to re-establish regular visitation and care coordination as the pandemic wanes, particularly for vulnerable seniors and those unable to independently access services [34, 35].

The findings from this study have several important implications for clinical practice and policy decisions regarding COVID-19 vaccination of older adults with dementia. First, we provide novel evidence that vaccination conferred significant protection against mortality even during the highly transmissible Omicron period. This adds to the scarce literature and reinforces vaccination as a critical strategy for protecting this vulnerable population.

Second, by applying ML models, we were able to generate personalized risk predictions based on individual patient characteristics. This offers clinicians valuable guidance to help identify those at highest risk of poor outcomes who may warrant more aggressive preventive measures or care planning. Such individualized risk stratification could help optimize resource allocation and clinical management of COVID-19 in this complex patient group [36].

Finally, at a policy level, our results provide public health decision-makers with real-world effectiveness data to inform vaccination prioritization, booster recommendations, and public messaging targeting older adults with dementia and their caregivers. Demonstrating the ongoing protection against severe illness and death enhances confidence in COVID-19 vaccines as a priority intervention for this high-risk population.

### Limitations

This study had several limitations. First, the single health system cohort may not fully generalize findings to other populations, though the large diverse sample provides valuable real-world data. Second, while rigorous methods were used to account for confounding, residual confounding from unmeasured factors is possible in this observational study. The analyses would be strengthened with additional details on timing of vaccination, specific vaccine types, and reasons for non-vaccination. Longer-term follow-up is needed to ascertain enduring protective effects. Further, while IPTW was used to balance observed factors, residual bias from unmeasured confounders not included in the propensity score model cannot be ruled out.

Nevertheless, leveraging a robust predictive modeling approach, this study offers clinically useful evidence that COVID-19 vaccination substantially reduces mortality risk in a vulnerable population of older adults with cognitive impairment, underscoring the importance of focused efforts to increase vaccine uptake among those at highest risk. It's possible some of those who remained unvaccinated represented a very high-risk group. Advanced health issues, disability, or severe cognitive impairment could have impacted their ability to consent to or access vaccination. For those with extreme frailty or dementia, the choice may have been out of their control. Therefore, the unvaccinated in our study may over-represent the oldest and sickest individuals faced with barriers to vaccination. We cannot rule out residual confounding from factors like diminished capacity, despite our adjustments. Generalizability to very frail older populations is limited.

## Conclusions

Our analysis reveals a significant impact of COVID-19 vaccination on reducing mortality risk within a large, demographically diverse cohort of older adults, both with and without cognitive impairment. Notably, among those diagnosed with cognitive disorders, vaccination was associated with a remarkable 93% (*p* < 0.001) survival rate. This rate was derived through a comparative analysis, between vaccinated and unvaccinated individuals within registar cognitive disorder subgroup, highlighting the profound protective effect of vaccination. These findings underscore the urgent need to enhance vaccination efforts and address disparities, ensuring vulnerable populations receive the protection they critically need to prevent mortality Additionally, the machine learning models, particularly XGBoost with inverse probability of treatment weighting, provide clinically useful tools to reliably predict individual patient mortality risk based on key factors like vaccination status, hospitalization duration, and dementia severity. Overall, the study highlights the vital importance of holistic, patient-centered medical care and equitable access to protective vaccination to improve outcomes for older adults during COVID-19.

### Supplementary Information

**Supplementary file 1.**

**Supplementary file 2.**

## Data Availability

The datasets used and/or analysed during the current study available from the corresponding author on reasonable request
