# Supplementary material for: Modeling mortality prediction in older adults with dementia receiving COVID-19 vaccination
Source: BMC Geriatr. 2024 May 24;24:454. doi: 10.1186/s12877-024-04982-7 (PMC11127399; doi:10.1186/s12877-024-04982-7)
Supplement: Supplementary file 1 [file 12877_2024_4982_MOESM1_ESM.pdf]

	Unvaccinated (N=16732)		Vaccinated (N=68556)		p values (chi test)
	Mean	SD	Mean	SD	
Age	79.35	6.30	77.16	5.66	<0.001
Socioeconomic class	10.33	3.99	11.51	4.32	<0.001
	n	%	n	%	
Male	7465	45	37904	55	<0.001
Female	9267	40	30652	62	<0.001
Registered in the Cognitive Disorders Registry	6329	38	17451	25	<0.001
Mortality	8481	51	2174	3	<0.001
Dementia	1275	8	4021	6	<0.001
Positive for COVID-19	2992	18	25353	37	<0.001
Utilization of antipsychotic drugs	4084	24	8460	12	<0.001
Prescription of antipsychotic drugs	2905	17	7548	11	<0.001
Utilization of antidepressants	6022	36	25585	37	<0.05
Prescription of antidepressants	5345	32	25507	37	<0.001
Depression diagnosis	3629	22	13442	20	<0.001
Home treatments	2866	17	2852	4	<0.001
Fractures	1731	10	10176	15	<0.001
Registered high blood pressure	12377	74	49153	72	<0.01
Registered COPD	1709	10	6190	9	<0.001
Registered diabetes	6249	37	22856	33	<0.001
Registered immunosuppression	4650	28	12155	18	<0.001
Registered obesity	2106	13	19836	29	<0.001
Nursing home entitlement	2450	15	3722	5	<0.001

eAppendix 1: Baseline Characteristics: A Comparison of Demographic Features, Comorbidities, and Healthcare Outcomes in Vaccinated and Unvaccinated Cohorts. Demographic characteristics, comorbidities, and healthcare outcomes of vaccinated and

unvaccinated individuals. Data was collected at the end of the T2 period, with the number of people (n) and percentage values presented in the table.
