# Supplementary material for: Modeling mortality prediction in older adults with dementia receiving COVID-19 vaccination
Source: BMC Geriatr. 2024 May 24;24:454. doi: 10.1186/s12877-024-04982-7 (PMC11127399; doi:10.1186/s12877-024-04982-7)
Supplement: Supplementary file 2 [file 12877_2024_4982_MOESM2_ESM.pdf]

Model	Weighted method	AUC [95% CI]	AUC-PR [95% CI]	TP/FP/FN/TN	Specificity	Sensitivity	Recall	Precision	F1 Score	Balanced Accuracy
**Logistic Regression**	None	0.9665 [0.9665-0.9665]	0.8393 [0.8393-0.8396]	12117/272/500/1269	0.7499	0.9455	0.7174	0.8235	0.7668	0.8477
**RandomForest**	None	0.9701 [0.97-0.9701]	0.8779 [0.8777-0.8779]	12205/184/500/1269	0.7508	0.9517	0.7174	0.8734	0.7877	0.8513
**XGBoost**	None	0.9763 [0.9762-0.9763]	0.8905 [0.8903-0.8905]	12176/213/474/1295	0.7634	0.9515	0.7321	0.8588	0.7904	0.8574
**LightGBM**	None	0.9757 [0.9757-0.9757]	0.8889 [0.8887-0.8889]	12166/223/466/1303	0.7672	0.9513	0.7366	0.8539	0.7909	0.8593
**CatBoost**	None	0.9751 [0.9751-0.9751]	0.8849 [0.8849-0.8851]	12202/187/511/1258	0.7453	0.9507	0.7111	0.8706	0.7828	0.8480
**Logistic Regression**	class_weight='balanced'	0.9673 [0.9673-0.9673]	0.824 [0.824-0.8243]	11072/1317/163/1606	0.9061	0.8955	0.9079	0.5494	0.6846	0.9008
**RandomForest**	class_weight='balanced'	0.9672 [0.9672-0.9673]	0.8698 [0.8696-0.8699]	12204/185/523/1246	0.7394	0.9500	0.7044	0.8707	0.7788	0.8447
**RandomForest**	'balanced_subsample'	0.9698 [0.9698-0.9699]	0.8771 [0.877-0.8772]	12208/181/520/1249	0.7410	0.9505	0.7060	0.8734	0.7809	0.8457
**XGBoost**	scale_pos_weight	0.9759 [0.9759-0.9759]	0.8858 [0.8857-0.8859]	11270/1119/134/1635	0.9224	0.9115	0.9243	0.5937	0.7230	0.9170
**LightGBM**	class_weight='balanced'	0.9757 [0.9756-0.9757]	0.8852 [0.8851-0.8853]	11315/1074/139/1630	0.9204	0.9143	0.9214	0.6028	0.7288	0.9174
**LightGBM**	is_unbalance	0.9749 [0.9748-0.9749]	0.8821 [0.8819-0.8821]	11310/1079/136/1633	0.9218	0.9142	0.9231	0.6021	0.7289	0.9180
**LightGBM**	scale_pos_weight	0.9749 [0.9748-0.9749]	0.8821 [0.882-0.8822]	11310/1079/136/1633	0.9218	0.9142	0.9231	0.6021	0.7289	0.9180
**CatBoost**	auto_class_weights='balanced'	0.9751 [0.9751-0.9751]	0.8821 [0.882-0.8822]	11271/1118/138/1631	0.9205	0.9113	0.9220	0.5933	0.7220	0.9159

**CatBoost**	auto_class_weights='SqrtBalanced'	0.9755 [0.9754-0.9755]	0.8866 [0.8866-0.8868]	11966/423/327/1442	0.8340	0.9470	0.8151	0.7732	0.7936	0.8905
**CatBoost**	scale_pos_weight	0.9751 [0.9751-0.9752]	0.8821 [0.882-0.8822]	11271/1118/138/1631	0.9205	0.9113	0.9220	0.5933	0.7220	0.9159
**Logistic Regression**	LogisticRegression propensity weighted	0.9652 [0.9651-0.9652]	0.8411 [0.841-0.8413]	12114/275/511/1258	0.7445	0.9445	0.7111	0.8206	0.7620	0.8445
**RandomForest**	LogisticRegression propensity weighted	0.9696 [0.9696-0.9697]	0.8767 [0.8766-0.8769]	12210/179/519/1250	0.7415	0.9507	0.7066	0.8747	0.7817	0.8461
**XGBoost**	LogisticRegression propensity weighted	0.9762 [0.9762-0.9762]	0.8901 [0.89-0.8902]	12182/207/477/1292	0.7620	0.9517	0.7304	0.8619	0.7907	0.8568
**LightGBM**	LogisticRegression propensity weighted	0.9759 [0.9759-0.9759]	0.8875 [0.8874-0.8876]	12181/208/476/1293	0.7624	0.9517	0.7309	0.8614	0.7908	0.8571
**CatBoost**	LogisticRegression propensity weighted	0.9754 [0.9754-0.9754]	0.8877 [0.8876-0.8878]	12194/195/480/1289	0.7606	0.9523	0.7287	0.8686	0.7925	0.8565
**Logistic Regression**	GaussianNB propensity weighted	0.9655 [0.9655-0.9656]	0.8351 [0.8351-0.8354]	12094/295/495/1274	0.7522	0.9442	0.7202	0.8120	0.7633	0.8482
**RandomForest**	GaussianNB propensity weighted	0.9699 [0.9699-0.97]	0.8774 [0.8773-0.8775]	12212/177/501/1268	0.7504	0.9521	0.7168	0.8775	0.7890	0.8513

**XGBoost**	GaussianNB propensity weighted	0.9761 [0.9761-0.9761]	0.8886 [0.8886-0.8888]	12188/201/488/1281	0.7566	0.9513	0.7241	0.8644	0.7881	0.8540
**LightGBM**	GaussianNB propensity weighted	0.9763 [0.9763-0.9764]	0.8904 [0.8903-0.8904]	12176/213/463/1306	0.7688	0.9523	0.7383	0.8598	0.7944	0.8605
**CatBoost**	GaussianNB propensity weighted	0.9755 [0.9755-0.9756]	0.8888 [0.8887-0.8889]	12199/190/476/1293	0.7626	0.9530	0.7309	0.8719	0.7952	0.8578

eAppendix 2: Performance Comparison of Various Machine Learning Models for Binary Mortality Prediction

Description: The table presents the performance metrics of different machine learning models used for binary mortality prediction. Models are evaluated based on AUC, AUC-PR, confusion matrix - TP/FP/FN/TN, specificity, sensitivity, recall, precision, F1 score, and balanced accuracy. The models include basic machine learning algorithms, models using built-in weighted imbalance methods, and models using inverse probability of treatment weighting with both Logistic Regression and GaussianNB.
